# Effects of galactooligosaccharides on maternal gut microbiota, glucose metabolism, lipid metabolism and inflammation in pregnancy: A randomized controlled pilot study

**DOI:** 10.3389/fendo.2023.1034266

**Published:** 2023-01-27

**Authors:** Jiayang Wan, Lin An, Zhenghong Ren, Shuxian Wang, Huixia Yang, Jingmei Ma

**Affiliations:** ^1^ Department of Obstetrics and Gynecology, Peking University First Hospital, Beijing, China; ^2^ Beijing Key Laboratory of Maternal Fetal Medicine of Gestational Diabetes Mellitus, Peking University First Hospital, Beijing, China; ^3^ Department of Maternal and Child Health, School of Public Health, Peking University, Beijing, China

**Keywords:** gut microbiota, pregnancy, galactooligosaccharides, prebiotic, gestational diabetes mellitus, metabolism

## Abstract

**Background:**

Gut microbiota of pregnant women change with the gestational week. On the one hand, they participate in the metabolic adaptation of pregnant women. On the other hand, the abnormal composition of gut microbiota of pregnant women is more likely to suffer from gestational diabetes mellitus (GDM). Therefore, gut microbiota targeted treatment through dietary supplements is particularly important for prevention or treatment. Prebiotic supplements containing galactooligosaccharides (GOS) may be an intervention method, but the effect is still unclear.

**Objective:**

This study aims to evaluate the feasibility and acceptability of prebiotic intervention in healthy pregnant women during pregnancy, and to explore the possible effects of intervention on pregnant women and the influence on gut microbiota as preliminaries.

**Methods:**

After recruitment in first trimester, 52 pregnant women were randomly assigned to receive GOS intervention or placebo containing fructooligosaccharides. 16S rRNA sequencing technology was used to detect the composition, diversity and differential flora of gut microbiota. Lipid metabolism, glucose metabolism and inflammatory factors during pregnancy were also analyzed.

**Results:**

The adverse symptoms of GOS intervention are mild and relatively safe. For pregnant women, there was no significant difference in the GDM incidence rates and gestational weight gain (GWG) in the GOS group compared with placebo (P > 0.05). Compared with the placebo group, the levels of FPG, TG, TC, HDL-C LDL-C, and IL-6 had no significant difference in GOS group (P > 0.05). For newborns, there was no significant difference between GOS group and placebo group in the following variables including gestational week, birth weight, birth length, head circumference, chest circumference, sex, and delivery mode (P > 0.05). And compared with the placebo group, the GOS group had a higher abundance of *Paraprevotella* and *Dorea*, but lower abundance of *Lachnospiraceae*UCG_001.

**Conclusions:**

GOS prebiotics appear to be safe and acceptable for the enrolled pregnancies. Although GOS intervention did not show the robust benefits on glucose and lipid metabolism. However, the intervention had a certain impact on the compostion of gut microbiota. GOS can be considered as a dietary supplement during pregnancy, and further clinical studies are needed to explore this in the future.

## 1 Introduction

With the change of gestational age, gut microbiota is participated in the physiological adaptation of maternal metabolism (1). Meanwhile, the abnormal composition of gut microbiota in pregnant women is related to the high possibility of complications during pregnancy, such as gestational diabetes mellitus (GDM) (2). The higher bacterial richness detected in GDM patients is also correlated with metabolic and inflammatory indicators (3). Some clinical trials suggested that intervention with dietary supplements during pregnancy may have different benefits for pregnant women (4). Some probiotics containing Lactobacillus or Bifidobacterium reduced the incidence of GDM to a certain extent (5). However, considering different intervention durations, strains, and doses, some studies did not support this view (6, 7). And other studies have shown that some dietary fiber can help alleviate type 2 diabetes (T2D) in non-pregnant people by regulating gut microbiota (8). Therefore, developing strategies to regulate gut microbiota is a potential direction to improve maternal metabolic health.

Different from probiotics, galactooligosaccharides (GOS) is a kind of prebiotics that aren’t digested and absorbed by the host, but can selectively promote the metabolism and proliferation of beneficial bacteria in the body, particularly by Lactobacillus and Bifidobacterium (9). GOS is a functional oligosaccharide with natural properties, and are composed of 3-10 molecules of galactose and glucose (10). GOS has the potential to protect against lipopolysaccharide (LPS) induced intestinal barrier injury (11). GOS can promote the increase of intestinal butyrate producing bacteria and promote the production of short-chain fatty acids (SCFAs) (12). SCFAs and G protein−coupled receptors 41/43 (GPR41/43) promote acute inflammatory responses in the intestine for tissue inflammation and protective immunity (13). GOS was also found to improve lipid metabolism in mice experiments (14). And for humans, GOS prebiotic supplements have certain effects on immune response. After prebiotics supplementation, pro-inflammatory cytokines interleukin-6 (IL-6), tumor necrosis factor-α (TNF-α), interleukin-1β (IL-1β) decreased, while anti-inflammatory cytokines interleukin-10 (IL-10) raised (15). GOS intervention can also increase *Bifidobacterium* that beneficial to human health (16). The use of prebiotics and other dietary supplements during pregnancy or lactation can produce beneficial gut microbiota in cesarean-delivered newborns, especially *Bifidobacterium* colonization (17).

Therefore, prebiotics have the potential to promote health and regulate gut microbiota. However, the beneficial effects of prebiotics during pregnancy remain unclear, the study of GOS prebiotics intervention on pregnant women is still in the preliminary exploration stage. This pilot randomized controlled pilot study aims to evaluate the feasibility, acceptability, and safety of prebiotic intervention for healthy pregnant women, and preliminarily explore the possible benefits for pregnant women.

## 2 Materials and methods

### 2.1 Study population

We conducted a prospective double-blinded randomized clinical trial involving singleton pregnancy women. Inclusion criteria were: 18-40 years of age; living in Beijing; understanding and willing to sign informed consent; singleton pregnancy; first prenatal care visit between 5-8 weeks of gestation. Exclusion criteria were: smoking, excessive alcohol or drug abuse; pregnancy complicated with chronic diseases (pre-existing diabetes, impaired glucose tolerance, impaired fasting glucose, chronic hypertension and so on); taken any prescribed chronic medications; steroids use.

The trial was recruited at Peking University First Hospital (PUFH), which is a public hospital located in Beijing, China. This study protocol has been approved by PUFH Clinical Trial Ethics Committee (reference number: 164). All patients provided written informed consent. The clinical trial was registered on www.chictr.org.cn (trial registration number: ChiCTR1800017192). The protocol of this study has been published online, which shows the whole recruitment process in detail (18). Our pilot RCT is conducted and reported in accordance with the Consolidated Standards of Reporting Trials guidelines for randomized pilot and feasibility trials (19). Recruitment commenced in August 2020 and finished in December 2021.

### 2.2 Study design and intervention

During this double-blinded, parallel-group clinical study, participants were randomly assigned to the control group and the intervention group at a 1:1 ratio. Women participants who meet the eligibility criteria were recruited and stratified according to their body mass index (BMI). All participants were divided into four groups underweight (BMI<18.5 kg/m^2^), normal weight (BMI 18.5–23.9 kg/m^2^) overweight (BMI 24–27.9 kg/m^2^) and obesity (BMI>28 kg/m^2^) (20). Computer-generated random numbers are used on the ‘H6WORLD’ platform (www.h6world.cn) to produce the randomized sequences. Based on BMI stratification, participants were automatically assigned to the control group or intervention group according to random sequences.

Subsequently, participants took GOS supplements in the intervention group or placebo containing fructooligosaccharides (FOS) in the control group from the first trimester (T1). In intervention group, GOS (6 g/100 g) and sialic acid (3 g/100 g) were the primary ingredients. The control group mainly contained FOS (3 g/100 g). The purities of GOS and FOS were 90% and 93% (w/w) on dry matter respectively. The dietary supplements were provided by the Beijing Sanyuan Foods Co. Ltd, Beijing, China. The dosage of the supplements was 60g per day. In order to improve pregnancy health care and strengthen adherence, both the two groups were provided with supplements containing nutrients, minerals and vitamins at each visit timepoint. The trial process followed the double-blind principle of researchers and participants.

### 2.3 Data and sample collection

Participants were enrolled at 5-8 weeks of gestation. Blood and stool samples were collected and followed up at 11-13 weeks of gestation and 24-28 weeks of gestation. During the follow-up period, filled in the questionnaire during the corresponding pregnancy, and left the participants’ blood samples and stool samples at two time points. All the 52 participants who were finally included in the study took blood samples and stool samples in both periods. All samples were collected in sterile tubes and stored at -80 °C until testing. The data of biochemical indexes such as glucose and lipid metabolism of pregnant women were obtained through the medical record system. After blood samples were collected, the immunological parameters IL-6 level was detected in the laboratory department. Fecal samples were collected for gut microbiota analysis.

### 2.4 Study outcomes

For the primary study outcomes, the effect of GOS on maternal gut microbiota were reported. At the same time, for those who have been followed up to the second trimester of pregnancy, based on the results of 75g oral glucose tolerance test (OGTT) at 24-28 gestational weeks (21), GDM incidence rates in these populations were reported.

For pregnant women, baseline data such as age, gravidity, parity, BMI, history of GDM, and family history of diabetes were described. For secondary outcomes, the biochemical parameters of glucose and lipid metabolism (fasting plasma glucose (FPG), triglyceride (TG), total cholesterol (TC), high-density lipoprotein cholesterol (HDL-C), low-density lipoprotein cholesterol (LDL-C)), IL-6, and gestational weight gain (GWG) in the intervention group and the control group were included respectively. As for Newborns, included gestational age, the mode of delivery, sex, birth length, birth weight, head circumference and chest circumference. We evaluated the safety and adverse reactions of prebiotics intervention.

### 2.5 DNA extraction and V3–V4 region of 16SrRNA gene sequencing

A commercial kit (Qiagen, Hilden, Germany) were used to extract faecal DNA. Faecal DNA was amplified by PCR using 16S amplicon PCR forward primer and 16S amplicon PCR reverse primer. After PCR amplification, the amplicons in each library were purified by Qiagen for library preparation. Subsequently, the qualified library was sequenced by Illumina Hiseq 2500 high-throughput sequencing platform. Sequences were clustered into operational taxonomic units (OTUs) based on Silva database v128, at a similarity level of 97%. Alpha and Beta diversity were generated in Quantitative Insights Into Microbial Ecology (QIIME). And the abundance of bacterial OTUs were divided into several levels (phyla, class, order, family and genus). The laboratory technicians were blinded to the clinical status (intervention or control group) of study participants.

### 2.6 Sample size

The purpose of this pilot study was to eavluate the feasibility and acceptability of prebiotics for pregnant women. A total of 52 pregnant women were considered enough to provide practical recruitment, feedback and compliance information. The findings will provide basis and support for future a large sample trial to evaluate the effects of prebiotics supplementation in early pregnancy on gut microbiota, glucose metabolism and immunity of pregnant women and newborns.

### 2.7 Statistical analysis

Data were represented as mean ± standard deviation (SD) or count (%). All data were input into SPSS (version 25.0) to analyze. GraphPad prism (version 8.0) was used to draw diagrams. χ2 and Fisher’s exact test was used for categorical variables, and t-test or non-parametric Wilcoxon test was used for continuous variables where appropriate. P < 0.05 was considered to be statistically significant. And bioinformatics analysis for microbiome used R software (Bell Laboratories). Alpha and beta diversities were generated in the Quantitative Insights Into Microbial Ecology (QIIME) and calculated based on weighted or unweighted Unifrac distance matrices. We used the linear discriminant analysis (LDA) effect size (LEfSe) method to identify species that show statistically significant differential abundances between groups.

## 3 Results

### 3.1 Participants enrollment and clinical baseline

**Figure 1:**
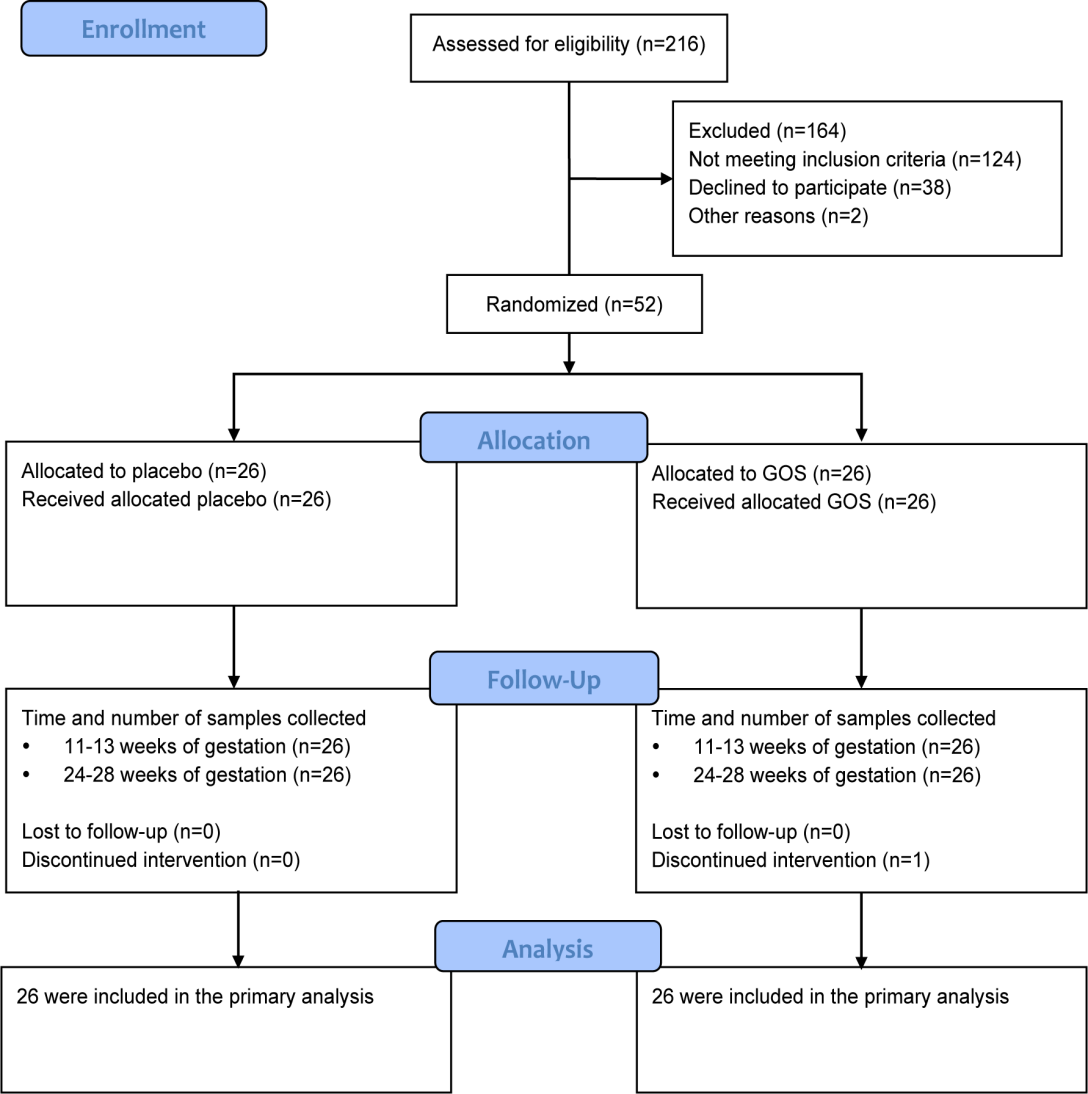
Flow chart of participants through the study.

Flow of participants through the study is shown in **Figure 1**. In total 216 women were assessed for eligibility. Of these, 124 did not meet the inclusion criteria, 38 declined the invitation to participate and 2 were excluded for other reasons. Fifty-two women were randomized, 26 to GOS and 26 to the placebo group. One woman in the GOS group withdrew early. Baseline characteristics were similar in GOS and placebo group, including age, height, pre-pregnancy weight, pre-pregnancy BMI, gravidity, parity, family history of diabetes, and history of GDM (**Table 1**).

**Table 1:** Baseline characteristics of study participants.

	GOS (n=26)	Placebo (n=26)	P-value
Age (years)	33.42 ± 3.71	32.35 ± 3.62	0.295
Height (cm)	164.04 ± 6.20	163.36 ± 5.64	0.682
Pre-pregnancy weight (kg)	59.23 ± 8.18	61.42 ± 11.90	0.444
Pre-pregnancy BMI (kg/m^2^)	22.05 ± 3.17	22.98 ± 4.20	0.368
BMI classification [n (%)]			0.914
Underweight (BMI<18.5 kg/m^2^)	1 (3.8)	2 (7.7)	
Normal weight (BMI 18.5–23.9 kg/m^2^)	20 (76.9)	18 (69.2)	
Overweight (BMI 24–27.9 kg/m^2^)	3 (11.5)	3 (11.5)	
Obesity (BMI>28 kg/m^2^)	2 (7.7)	3 (11.5)	
Gravidity	0.65 ± 0.94	0.92 ± 1.16	0.362
Parity	0.23 ± 0.43	0.27 ± 0.53	0.776
Family history of diabetes [n (%)]			0.191
Yes	5 (19.2)	1 (3.8)	
No	21 (80.8)	25 (96.2)	
History of GDM [n (%)]			1.000
Yes	0 (0.0)	1 (3.8)	
No	26 (100.0)	25 (96.2)	

Data presented are mean ± SD or n (%).

P-values for comparisons between the 2 groups in t-tests for continuous variables, and χ2 and Fisher’s exact tests for categorical variables.

GDM, gestational diabetes mellitus; BMI, body mass index.

### 3.2 Effects of prebiotics on gut microbiota in pregnant women

#### 3.2.1 Overall microbial structures of gut microbiota

**Figure 2:**
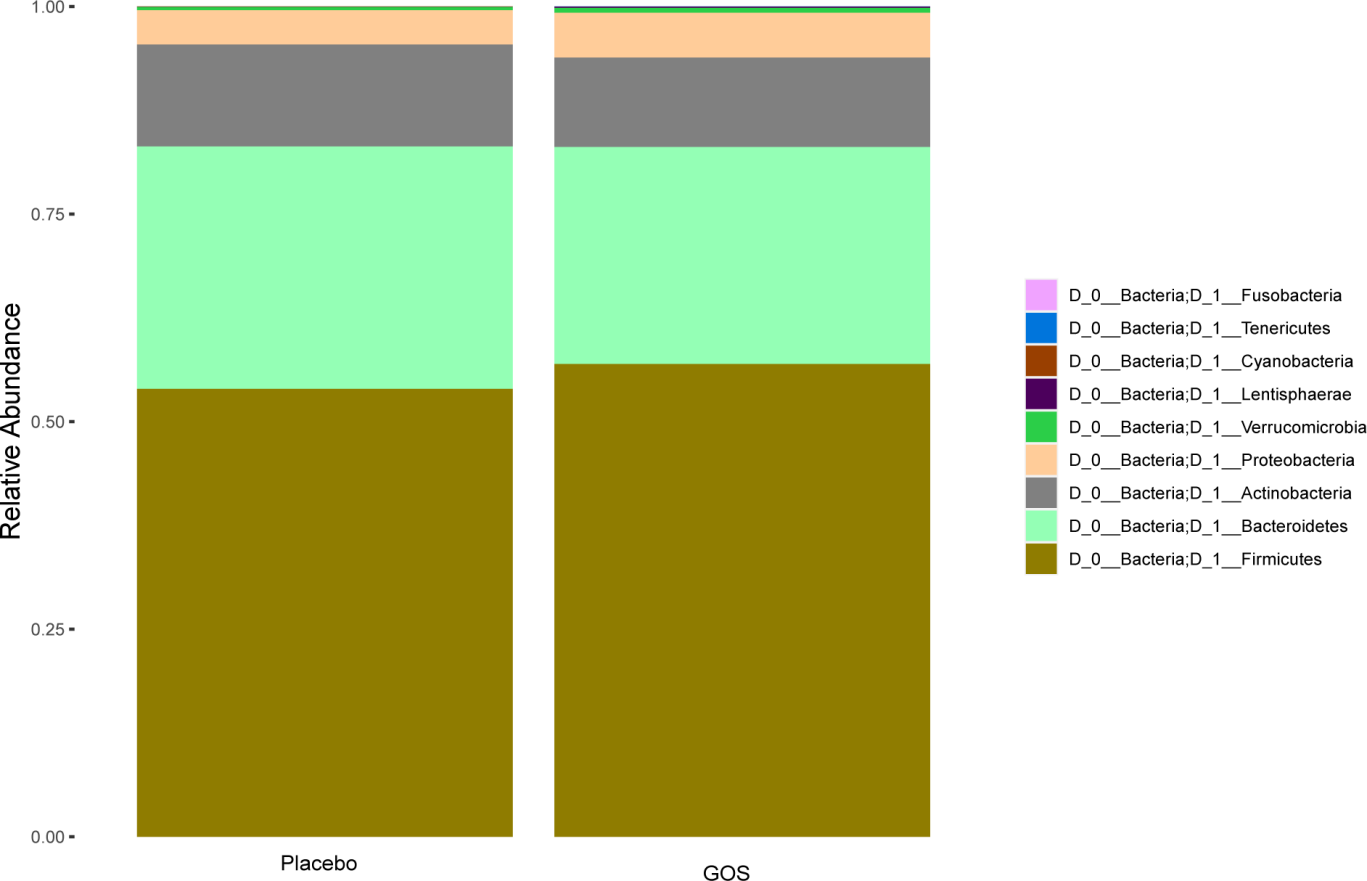
Relative abundance at the level of bacterial phylum.

We studied gut microbiota of women in placebo and GOS groups. **Figure 2** shows the overall microbiota structure at the phylum level in each group. The main phyla of placebo and GOS groups were *Firmicutes*, *Bacteroidetes*, *Actinobacteria*, and *Proteobacteria*, with *Firmicutes* the most abundant.

#### 3.2.2 Changes of gut microbiota diversity

**Figure 3:**
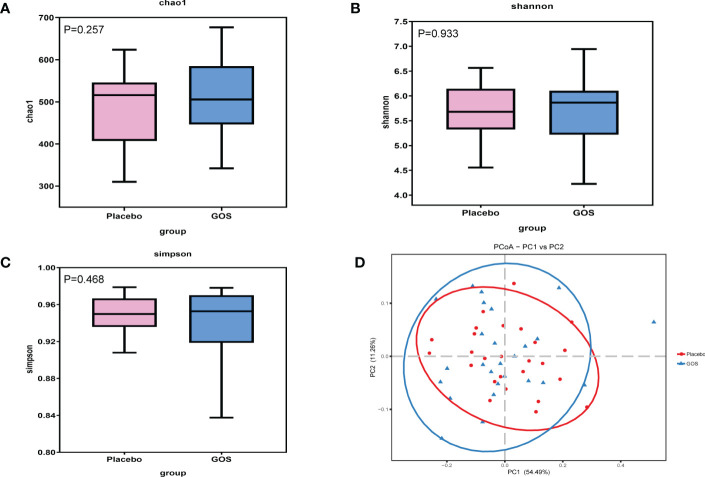
Alpha and beta diversity of gut microbiota in placebo and GOS groups. **(A)** Comparisons of Chao 1 index. **(B)** Comparisons of Shannon’s index. **(C)** Comparisons of Simpson’s index. **(D)** PCoA calculated based on Weighted unifrac distances.

To assess the gut microbiota community structure, richness (Chao 1 index) and diversity (Simpson index, Shannon index) were calculated (**Figures 3A–C**). There was no significant difference in Chao 1 index between GOS group and placebo group (P > 0.05). For Simpson index and Shannon index, compared with placebo group, the data of the GOS group were similarly (P > 0.05).

To compare overall gut microbiota structure in pregnant women, PCoA according to OTUs of each sample were implemented to provide a glimpse of gut microbial dynamics between placebo and GOS groups. The results of PCoA were PC1 = 54.49% and PC2 = 11.26% of total variations (**Figure 3D**).

#### 3.2.3 Changes in specific bacterial taxa

**Figure 4:**
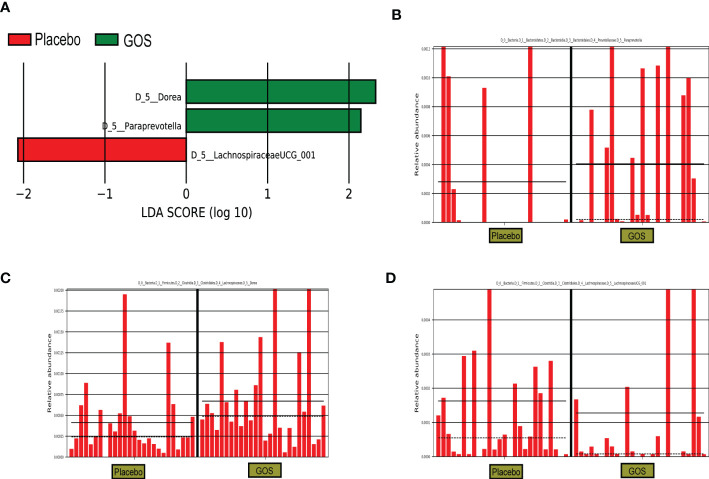
Identification of the most differentially abundant analyzed by the LEfSe method. **(A)** LDA scores of differentially abundant taxa. **(B)** Relative abundance of Paraprevotella. **(C)** Relative abundance of Dorea. **(D)** Relative abundance of LachnospiraceaeUCG_001.

For identify the changes in specific bacterial taxa after prebiotics supplemented intervention. We utilized the linear discriminant analysis (LDA) effect size (LEfSe) to compare the gut microbiota composition between placebo and GOS groups. The LDA score was selected to discriminate specific taxa in two groups. Compared with the placebo group, the GOS group had a higher abundance of *Paraprevotella* and *Dorea*, but lower abundance of *Lachnospiraceae*UCG_001 (**Figures 4A–D**).

### 3.3 Participants clinical outcomes

#### 3.3.1 GDM diagnosis and OGTT values

Serum levels of FBG, 1-hour and, 2-hour OGTT plasma glucose measured at 24–28 weeks of pregnancy in women who received either GOS or placebo are illustrated in **Table 2**. As can be seen, there was no significant difference between the intervention and the control group regarding FBG (4.75 ± 0.30 mmol/L vs 4.73 ± 0.41 mmol/L; P = 0.883), OGTT-1 h (7.81 ± 1.55 mmol/L vs 8.57 ± 2.03 mmol/L; P = 0.133), and OGTT-2 h (6.57 ± 1.44 mmol/L vs 6.87 ± 1.33 mmol/L; P = 0.434) measured at 24–28 weeks of pregnancy. The incidence of GDM in the GOS and placebo group are provided in **Table 2**. The incidence of GDM in the GOS group was 30.8% which was not significantly different from the placebo group (30.8%) (P = 1.000).

**Table 2:** GDM diagnosis and OGTT values.

	GOS (n=26)	Placebo (n=26)	P-value
Plasma glucose in OGTT (mmol/L)			
Fasting	4.75 ± 0.30	4.73 ± 0.41	0.883
1h	7.81 ± 1.55	8.57 ± 2.03	0.133
2h	6.57 ± 1.44	6.87 ± 1.33	0.434
GDM diagnosis [n (%)]			1.000
Yes	8 (30.8)	8 (30.8)	
No	18 (69.2)	18 (69.2)	

GDM, gestational diabetes mellitus; OGTT, oral glucose tolerance test.

#### 3.3.2 Changes in weight and BMI during pregnancy

With the change of gestational weeks, we collected the weight gain during pregnancy of two groups of pregnant women, and calculated the changes of BMI (**Table 3**). There was no significant difference in these indicators between GOS group and placebo group regarding gestational weight gain (GWG) (12.42 ± 3.63 kg vs 13.19 ± 3.94 kg; P = 0.466), BMI gain (4.65 ± 1.51 kg/m^2^ vs 4.93 ± 1.44 kg/m^2^; P = 0.497).

**Table 3:** Changes in weight and BMI during pregnancy.

	GOS (n=26)	Placebo (n=26)	P-value
BW 1st (kg)	59.23 ± 8.18	61.42 ± 11.90	0.444
BMI 1st (kg/m2)	22.05 ± 3.17	22.98 ± 4.20	0.368
GWG (kg)	12.42 ± 3.63	13.19 ± 3.94	0.466
BW 3rd (kg)	71.65 ± 8.96	74.61 ± 13.43	0.355
BMI 3rd (kg/m2)	26.70 ± 3.71	27.91 ± 4.66	0.303
BMI gain (kg/m2)	4.65 ± 1.51	4.93 ± 1.44	0.497

BW, body weight; BMI, body mass index; BW 1st, body weight at the beginning of 1st trimester; BW 3rd, body weight at the end of 3rd trimester; BMI 1st, BMI at the beginning of 1st trimester;

BMI 3rd, body mass index at the end of 3rd trimester; GWG, gestational weight gain.

#### 3.3.3 Clinical characteristics of neonates

To investigate the impact of the intervention on neonatal outcomes, we measured the following variables including gestational week, birth weight, birth length, head circumference, chest circumference, sex, and delivery mode. No significant difference was found between the GOS and placebo group (all P-values were > 0.05) (**Table 4**).

**Table 4:** Clinical characteristics of neonates.

	GOS (n=26)	Placebo (n=26)	P-value
Gestational week (weeks)	38.95 ± 1.58	38.86 ± 1.51	0.838
Birth weight (g)	3242.12 ± 484.20	3235.38 ± 443.53	0.959
Birth length (cm)	49.77 ± 1.53	49.65 ± 1.32	0.773
Head circumference (cm)	33.88 ± 0.44	33.94 ± 0.45	0.621
Chest circumference (cm)	32.64 ± 0.57	32.79 ± 0.57	0.356
Sex [n (%)]			0.578
Male	13 (50.0)	11 (42.3)	
Female	13 (50.0)	15 (57.7)	
Delivery mode [n (%)]			0.080
Spontaneous delivery	20 (76.9)	14 (53.8)	
Cesarean delivery	6 (23.1)	12 (46.2)	

#### 3.3.4 Glucose metabolism, lipid metabolism and inflammatory factor levels

In order to further explore the effect of prebiotics intervention on glucose metabolism, lipid metabolism, and immunity, we analyzed the following indicators (**Table 5**). There was no significant difference in glucose metabolism levels between GOS group and placebo group regarding FPG (4.75 ± 0.30 mmol/L vs 4.73 ± 0.41 mmol/L; P = 0.883). Meanwhile, there was no significant difference in lipid metabolism levels between GOS group and placebo group regarding TG (1.93 ± 0.75 mmol/L vs 2.05 ± 0.87 mmol/L; P = 0.615), TC (5.09 ± 1.27 mmol/L vs 5.20 ± 1.17 mmol/L; P = 0.737), HDL-C (1.56 ± 0.35 mmol/L vs 1.61 ± 0.37 mmol/L; P = 0.610), and LDL-C (2.65 ± 0.89 mmol/L vs 2.64 ± 0.80 mmol/L; P = 0.983). There was also no significant difference in IL-6 levels between GOS group and placebo group (1.55 ± 0.58 pg/mL vs 2.02 ± 1.20 pg/mL; P = 0.080).

**Table 5:** Glucose metabolism, lipid metabolism and inflammatory factor levels.

	GOS (n=26)	Placebo (n=26)	P-value
FPG (mmol/L)	4.75 ± 0.30	4.73 ± 0.41	0.883
TG (mmol/L)	1.93 ± 0.75	2.05 ± 0.87	0.615
TC (mmol/L)	5.09 ± 1.27	5.20 ± 1.17	0.737
HDL-C (mmol/L)	1.56 ± 0.35	1.61 ± 0.37	0.610
LDL-C (mmol/L)	2.65 ± 0.89	2.64 ± 0.80	0.983
IL-6 (pg/mL)	1.55 ± 0.58	2.02 ± 1.20	0.080

FPG, fasting plasma glucose; TG, triglyceride; TC, total cholesterol; HDL-C, high-density lipoprotein cholesterol; LDL-C, low-density lipoprotein cholesterol; IL-6, interleukin-6.

#### 3.3.5 Incidences of maternal and infant complications

Clinical data on the incidences of maternal and infant complications were also collected (**Table 6**). The incidence of gestational hypertension was 0.0% in the GOS group and 11.5% in the placebo group (P = 0.235). The incidence of thyroid dysfunction was 3.8% in the GOS group and 15.4% in the placebo group (P = 0.350). The incidence of fetal growth restriction was 3.8% in the GOS group and 0.0% in the placebo group (P = 1.000). The incidence of anemia was 34.6% in the GOS group and 42.3% in the placebo group (P = 0.569). And the incidence of postpartum hemorrhage was 7.7% in the GOS group and 3.8% in the placebo group (P = 1.000).

**Table 6:** Incidences of maternal and infant complications.

	GOS (n=26)	Placebo (n=26)	P-value
Gestational hypertension [n (%)]	0 (0.0)	3 (11.5)	0.235
Thyroid dysfunction [n (%)]	1 (3.8)	4 (15.4)	0.350
Fetal growth restriction [n (%)]	1 (3.8)	0 (0.0)	1.000
Anemia [n (%)]	9 (34.6)	11 (42.3)	0.569
Postpartum hemorrhage [n (%)]	2 (7.7)	1 (3.8)	1.000

### 3.4 Safety of intervention

A questionnaire was used to record the possible severity of adverse symptoms in pregnant women and the relationship between symptoms and the ingestion of preparations. The results showed that one participant in GOS group had abdominal distension and one participant had nausea. These symptoms have little to do with the intake of prebiotic preparations, and may be related to appetite and hormone changes during pregnancy. Therefore, for the existing included cases, it can be considered that supplementing prebiotic preparations during pregnancy is relatively safe.

## 4 Discussion

During pregnancy, the disorder of gut microbiota and abnormal glucose metabolism may be the possible mechanism of pregnancy complications such as GDM (22). Moreover, patients with gestational diabetes have a higher chance of developing type 2 diabetes in the long term (23). Maternal GDM is also associated with overweight and obesity status in offspring (24). Therefore, it is necessary to seek safe and effective interventions to improve the adverse status of pregnant women. There have been studies on the use of probiotics, synbiotics and other dietary supplements during pregnancy to prevent and treat gestational diabetes (5, 25). The purpose of this study is to explore the effects of prebiotic preparations containing GOS on glucose metabolism, lipid metabolism, inflammation and gut microbiota of pregnant women during early pregnancy, and the feasibility and acceptability of using prebiotics as dietary supplements during pregnancy.

The preliminary conclusion of this study is that GOS intervention has no significant effect on reducing the incidence of GDM and improving glucose and lipid metabolism. GOS a kind of prebiotics that can be selectively and selectively utilized by host microorganisms that confer a health benefit, while probiotics are defined as live microorganisms (26). Previous clinical studies using probiotic supplements for intervention have some similarities with this study, the results showed that probiotics did not reduce the incidence of GDM in pregnant women (6, 7, 27), there is a study with different conclusion (5). There were no significant changes in FBG and insulin resistance index for some synbiotics containing fructooligosaccharide (28). An animal study in GDM mice showed that inulin-type fructose-oligosaccharide treatment alleviated glucose and lipid metabolism disorders mediated by the gut microbiota (29). Dietary supplements intervention may be beneficial in improving inflammatory status. A clinical trial supplemented with probiotics also observed that dietary supplements reduced the expression of pro-inflammatory factors TNF-α (30). However, as far as we know, there are few clinical studies on prebiotic supplements for pregnant women. The different results may be related to the type, dose, dosage form, intervention time and intervention population of dietary supplements. Our intervention seems to be safe and well tolerated in view of the minimal adverse reactions. A meta-analysis also thought that probiotics and prebiotics are safe during pregnancy and lactation, and adverse reactions related to the use of probiotics and prebiotics will not cause any serious health problems to mothers or infants (31). In this study, GOS supplementation was started in early pregnancy. GOS may have some effect as the duration of the intervention increases if taken before pregnancy or even earlier. From the follow-up, participants gave us feedback that taking such dietary supplements was convenient and easy to implement. Prebiotics can usually be added to common foods, they are mainly used for fermented dairy products (yogurt, cheese), nonfermented dairy products (milk formula for pregnant women or infant) (9). Therefore, in addition to the need to supplement essential nutrients during pregnancy, it is also possible for pregnant women to take appropriate food containing prebiotics. At the same time, the clinical conditions of the pregnant women themselves and their daily energy intake should also be considered.

Both the intervention group and the control group have similar relative abundances at the phylum level, including *Firmicutes, Bacteroidetes, Actinobacteria, and Proteobacteria*. *Paraprevotella* and *Dorea* were enriched in the intervention group during the second trimester, suggesting that prebiotics could affect the composition of gut microbiota. A study has shown that the abundance of *Paraprevotella* is negatively correlated with serum TG, TC and LDL-C levels, suggesting that *Paraprevotella* may have anti-obesity effects (32). Prebiotics are not digested and absorbed by the host, but can promote the proliferation of target flora and improve intestinal microecology by increasing the abundance of beneficial bacteria in the intestine (33). In our work, after the intervention of GOS prebiotics, the relative abundance of *Paraprevotella* and *Dorea* increased specifically, and the relative abundance of *Lachnospiraceae*UCG_001 was higher in the placebo group containing FOS prebiotics. Although in our study, after the GOS intervention, some serum indicators, such as TG, LDL, etc. had no beneficial effects, but the intervention did not seem to have some adverse effects on these indicators. Based on the limited participants and the individual differences among pregnant women, it is necessary to explore the effect of prebiotics on serum indicators in the future.

Several strengths and limitations should be taken into consideration. First, this study is a randomized controlled pilot trial. Subsequently, 16S rRNA gene was sequenced by Illumina Hiseq 2500 sequencing platform, a widely and reliable used high-throughput sequencing platform, which can ensure gut microbiota can be successfully identified. Secondly, the quality control in the process of sample collection can be guaranteed, which makes the sequencing quality high and accurate. However, some limitations should also be considered. The sample size of our pilot study is limited, and some confounding factors such as diet and exercise have caused some interference. Although it is difficult to control these confounding factors, we recorded these situations in the form of health education and questionnaire records. Moreover, this study was recruited in the same hospital, and the potential regional differences of microbiota cannot be evaluated. In general, our study provides an important basis for the intervention of prebiotic dietary supplements targeting gut microbiota in pregnancy on metabolic diseases of pregnancy. In the future, clinical trials with higher quality and larger sample size are needed to further verify the effect of prebiotic supplements.

## 5 Conclusion

GOS prebiotics appear to be safe and acceptable for the enrolled pregnancies. Although GOS intervention did not show the robust benefits on glucose and lipid metabolism. However, the intervention had a certain impact on the compostion of gut microbiota. GOS can be considered as a dietary supplement during pregnancy, and further clinical studies are needed to explore this in the future.

## Data availability statement

The raw sequence data of the 16S rRNA gene supporting the results of this article are available in the NCBI database, SRA data (Accession number: PRJNA925813).

## Ethics statement

The studies involving human participants were reviewed and approved by Clinical Trial Ethics Committee, Peking University First Hospital, Beijing, China. The patients/participants provided their written informed consent to participate in this study.

## Author contributions

JW collected the data, prepared tables and figures, and drafted the paper. LA and ZR analyzed the data and prepared tables. SW, LA, HY, and JM conceived and designed the research. JM revised the manuscript. HY and JM provided clinical supervision. All authors contributed to the article and approved the submitted version.
